# Causal mechanism of injection-induced earthquakes through the M_w_ 5.5 Pohang earthquake case study

**DOI:** 10.1038/s41467-020-16408-0

**Published:** 2020-05-26

**Authors:** I. W. Yeo, M. R. M. Brown, S. Ge, K. K. Lee

**Affiliations:** 1 Department of Geological and Environmental Sciences, Chonnam National University, Yongbong-ro 77, Buk-gu, Gwangju, 61186 Republic of Korea; 2 Department of Geology and Environmental Geosciences, Northern Illinois University, 1425 W. Lincoln Hwy., DeKalb, IL 60115 USA; 3 Department of Geological Sciences, University of Colorado, Boulder, CO 80309-0399 USA; 4 School of Earth and Environmental Sciences, Seoul National University, 1 Gwanak-ro, Gwanak-gu, Seoul, 08826 Republic of Korea

**Keywords:** Geophysics, Seismology, Geothermal energy

## Abstract

Causal mechanisms for fluid injection-induced earthquakes remain a challenge to identify. Past studies largely established spatiotemporal correlations. Here, we propose a multi-process causal mechanism for injection-induced earthquakes through a case study of the 2017 M_w_ 5.5 induced earthquake near Pohang Enhanced Geothermal System, Korea, where detailed hydraulic stimulation and on-site seismicity monitoring data provide an unprecedented opportunity. Pore pressure modeling reveals that pore pressure changes initiate seismicity on critically stressed faults and Coulomb static stress transfer modeling reveals that earthquake interactions promote continued seismicity, leading to larger events. On the basis of these results, we propose the following causal mechanism for induced seismicity: pore pressure increase and earthquake interactions lead to fault weakening and ultimately triggering larger earthquakes later in the process. We suggest that it is prudent that pore pressure change, initial seismicity locations, and Coulomb static stress transfer from seismicity earlier in the sequence are assessed in real-time.

## Abstract (web summary)

The authors here suggest a causal mechanism for injection-induced earthquakes. They further suggest pore pressure modeling as a practical alternative to direct in-situ pore pressure observation which can then be used for stress build-up monitoring.

## Introduction

Disposal of wastewater through deep injection has resulted in a robust increase in induced seismicity^1^. Enhanced geothermal systems (EGS) also have a history of induced seismicity that resulted in either shutting down or disruption of operations. At Basel, Switzerland, a local magnitude (M_L_) 3.4 event occurred shortly after one of the hydraulic stimulations in 2006 and the geothermal project was shut down immediately afterwards^2,3^. At Soultz-sous-Forêts geothermal plant, France, a magnitude 2.9 event caused by hydraulic stimulations raised major concerns and led to modifications of stimulation operation^4,5^.

Correlations between seismicity and fluid injections abound^6–8^. Causal mechanisms, however, are sparse and often inconclusive. Elsworth et al. ^9^ summarized two types of causal mechanisms for inducing fault failure. One is due to pore pressure diffusion influence in regions around the injection as pore pressure reduces fault shear strength. The other is due to rock stress increasing which destabilizes faults in regions beyond the pore pressure influence^10^. Pore pressure increase reduces the effective stress on a fault and consequently the shear strength. Decreased effective normal stress causing fault failure is the classic view that originated from studying faulting^11^ and has since been used to explain injection-induced seismicity^12,13^. Poroelastic stress change from injection has also been evoked to explain seismicity at farther distances from injection where pore pressure influence is relatively small^14^. In-situ experiments have demonstrated that aseismic slips can create stresses that could trigger earthquakes beyond the spatial extent of pore pressure diffusion^15^ and such aseismic slip stress could propagate faster than pore pressure diffusion^16^. Cumulative Coulomb stress changes from smaller earthquakes on a fault can match or exceed the values of pore pressure increase and therefore become the triggering mechanisms^17,18^. Coulomb static stress can be modified to take into consideration pore pressure effects by replacing the friction coefficient with an effective friction coefficient^19,20^, in which pore pressures were estimated based on mean volumetric strain on faults but do not change with time. While these processes each contribute to triggering earthquakes, here we propose a multi-process causal mechanism for induced seismicity that has broad implications to how injection, wastewater disposal or hydraulic fracturing, induces seismicity. We use a case study where there is a high-resolution dataset available for establishing the basic elements of the proposed causal mechanism.

**Fig. 1:**
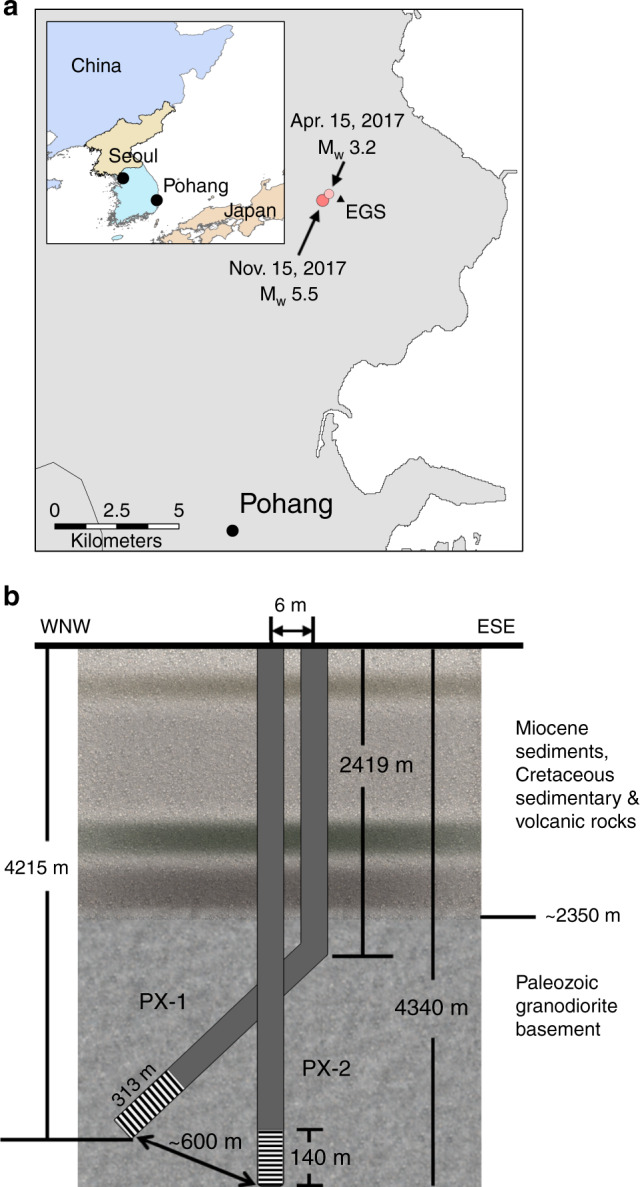
Pohang enhanced geothermal system. **a** Location of the Pohang EGS site, epicenters of the two largest events, April 15, 2017 M_w_ 3.2 and November 15, 2017 M_w_ 5.5. Inset shows the location of Pohang in South Korea. Country outlines from www.gadm.org. **b** Schematic diagram of two geothermal exploration wells, PX-1 and PX-2, and generalized lithology of the area^21^.

**Fig. 2:**
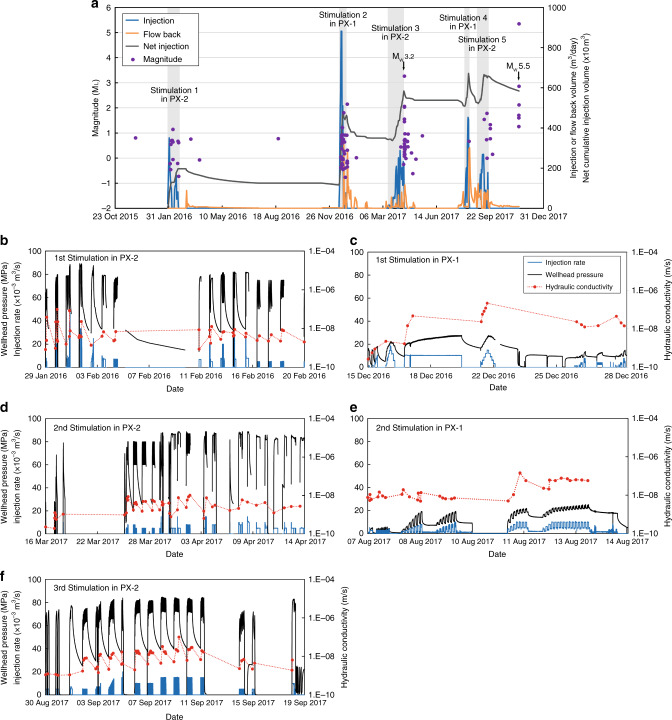
Stimulation data and seismicity. **a** Timeline of earthquakes and five hydraulic stimulations^21^: injection rate (blue), flow back rate (orange), and net injection volume (dark gray). The M_w_ 3.2 event occurred during flow back of the third stimulation and the M_w_ 5.5 event after the fifth stimulation. Here, flow back refers to fluids flowing back to ground surface driven by the pressure difference between the deep rock formation near the injection interval and the ground surface. The first, third, and fifth stimulations were conducted in PX-2 and the second and fourth in PX-1. **b–f** Wellhead pressure (black) and injection rate (blue) measured during five hydraulic stimulations, as well as hydraulic conductivity values (red) estimated by the analytical method using pressure and rate data. **b**, **d**, and **f** correspond to the first, third, and fifth stimulations (first, second, and third in PX-2). **c** and **e** correspond to the second and fourth stimulations (first and second in PX-1). Source data are provided as a Source Data file.

Pohang, Korea experienced a moment magnitude (M_w_) 5.5 earthquake on November 15, 2017. The epicenter was ~510 m from the Pohang EGS (Fig. 1a). A series of five hydraulic stimulations at the Pohang EGS took place from January 29, 2016 to September 18, 2017 in two exploratory wells (Fig. 1b) drilled into crystalline basement (>4 km depth). Each stimulation generated a swarm of seismicity that led to an M_w_ 3.2 event on April 15, 2017 and M_w_ 5.5 mainshock on November 15, 2017, two months after stimulation ceased (Fig. 2a).

Close correlations between hydraulic stimulations and seismicity over a period of 2 years and in-depth seismological analysis led to the conclusion that the M_w_ 5.5 earthquake was induced^21^. Spatiotemporal correlations between stimulation and seismicity are important steps in understanding a potential linkage between the two. Correlations have identified important characteristics of operation, such as injection rate as leading reasons for causing induced seismicity^6^. More sophisticated geospatial analyses correlated geographic centroids of injection wells and injection volumes to that of earthquakes, which has implications to guide future mitigation^7^. Equally central is the underlying causal mechanisms, i.e., the physical processes that take place through time and lead to a mainshock. In addition, the delayed mainshock occurrence at Pohang and at other sites continue to attract research efforts. For example, Sumy et al.^20^ suggested that Coulomb stress transfer from earthquakes earlier in the sequence could trigger larger events later. Langenbruch and Shapiro^22^ suggested that fracture strength is an important factor in controlling the post-injection delay rate and the existence of unstable fractures enhance the possibility of larger magnitude events. The detailed stimulation data, pressure modeling, and seismicity sequence supported a mechanism hypothesis that encompasses several processes identified in prior studies, which includes pore pressure diffusion, fault weakening, and static stress transfer. These processes are more likely working in concert rather than in isolation.

The objective of this study is to establish a synthesized causal mechanism for the mainshock by using the injection data to model pore pressure changes, relating pore pressure changes to seismicity occurrence, computing the Coulomb static stress from the earthquakes prior to the mainshock, and connecting these prior processes to the mainshock.

## Results

### Hydraulic diffusivity

To quantitatively examine spatiotemporal pore pressure changes following hydraulic stimulations of two wells (PX-1 and PX-2) at the Pohang EGS site, a numerical model based on pore pressure diffusion is developed. The key hydrologic parameter in modeling pore pressure diffusion is hydraulic diffusivity (*D*) defined as the ratio of hydraulic conductivity (*K*) and specific storage (*S*_s_). Hydraulic conductivity (*K*) is defined as *k*ρg/*μ*, where *k* is permeability, ρg is the unit weight of water, and *μ* is viscosity of water.

**Fig. 3:**
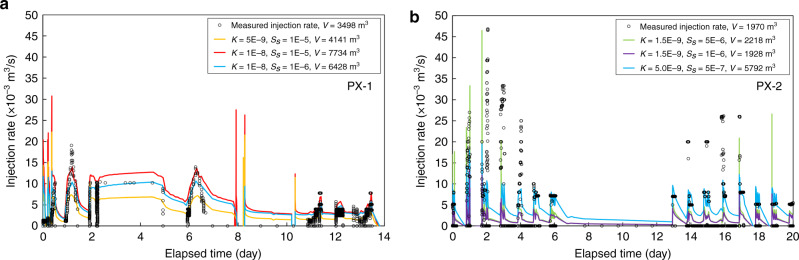
Numerical model calibration of hydraulic diffusivity. Wellhead pressures are model input. Modeled and measured flow rate at the injection sites and the net flow volume are compared for **a** the second (total) stimulation in PX-1 and **b** the first stimulation in PX-2. All combinations of *K* and *S*_s_ that led to a reasonable match between modeled (solid lines) and measured data (circles) are shown. These combinations cover a range of hydraulic diffusivities as summarized in Table 1. Source data are provided as a Source Data file.

An analytical method^23^ was used to derive hydraulic conductivity, and numerical modeling was used to derive hydraulic diffusivity (see Methods section). Wellhead pressure data obtained from repeated injection and flow back cycles during stimulations provide valuable data for inferring hydraulic conductivity around the injection wells. We utilized every episode of pressure build-up and drawdown as single-well tests to compute hydraulic conductivity. Single-well test data are not well suited for deriving specific storage; we use specific storage values from laboratory tests of the Pohang EGS 4.2 km deep granitic rock core^24^. Data from PX-2 stimulations yield a hydraulic conductivity range of 2.0 × 10^−10^ to 1.0 × 10^−7^ m s^−1^ (Fig. 2b, d, and f). There was no noticeable sustained increase in hydraulic conductivity after hydraulic stimulations. PX-1 stimulation data yield a hydraulic conductivity range of 3.0 × 10^−10^–2.0 × 10^−7^ m s^−1^ (Fig. 2c, e). Numerical modeling sensitivity analysis using wellhead pressure as input and injection rate as model constraints yield a hydraulic diffusivity range of 5.0 × 10^−4^– 1.0 × 10^−2^ m^2^ s^−1^ when using PX-1 flow rate as a constraint and 3.0 × 10^−4^–1.0 × 10^−2^ m^2^ s^−1^ when using PX-2 flow rate as a constraint (Fig. 3). Values of hydraulic conductivity and hydraulic diffusivity are summarized in Table 1.

**Table 1:** Summary of hydraulic conductivity and diffusivity for rocks around PX-1, PX-2, and a fault zone.

	Hydraulic conductivity, *K* (m s^−1^)	Hydraulic diffusivity, *D* (m^2^ s^−1^)^a^
PX-1, analytical Jacob method	3.0 × 10^−10^–2.0 × 10^−7^	6 × 10^−4^–4 × 10^−1^
PX-2, analytical Jacob method	2.0 × 10^−10^–1.0 × 10^−7^	4 × 10^−4^–2 × 10^−1^
PX-1, numerical model calibrated	–	5 × 10^−4^ to 1 × 10^−2^
PX-2, numerical model calibrated	–	3 × 10^−4^ to 1 × 10^−2^
PX-1^21^	2.0 × 10^−8^	4 × 10^−2^
Fault core, lab test^38^	1.0 × 10^−13^–1.0 × 10^−12^	1 × 10^−8^–1 ×10^−7^
Fault damage zone, lab test^38^	1.0 × 10^−6^–1.0 × 10^−4^	1 × 10^−1^–10

^a^Specific storage values (*S*_s_) of 5.0 × 10^−7^ m^−1^ for basement rock and 1.0 × 10^−5^ m^−1^ for the fault, estimated from laboratory mechanical tests, were used to calculate initial *D* from *K*.

Scale effects on hydrologic parameters are known to vary with measuring methods, with smaller values from core sample measurements and larger values from regional scale model calibrations^25^. Values from laboratory tests on core samples can be two or three orders of magnitude smaller than in-situ values at formation scales^26^. Hydraulic conductivity values typically increase as rock volume tested increases, allowing inclusion of fractures and geologic discontinuities in the tested volume. Synthesizing results from the analytical method and numerical modeling and considering scale effects, we obtain a hydraulic diffusivity of 0.01 m^2^ s^−1^ for the basement.

Analyzing pressure data during stimulations and recoveries suggested no notable changes in permeability under applied wellhead pressures up to 15 MPa at PX-1 and 65 MPa at PX-2, which indirectly suggests that the injection induced property changes in the formation and faults were neither substantial nor sustained. Above these wellhead pressures, permeability temporarily increased due to the opening of fractures. Repeated hydraulic stimulations however did not produce the overall increase of permeability (Fig. 2).

### Fault hydraulic characteristics

**Fig. 4:**
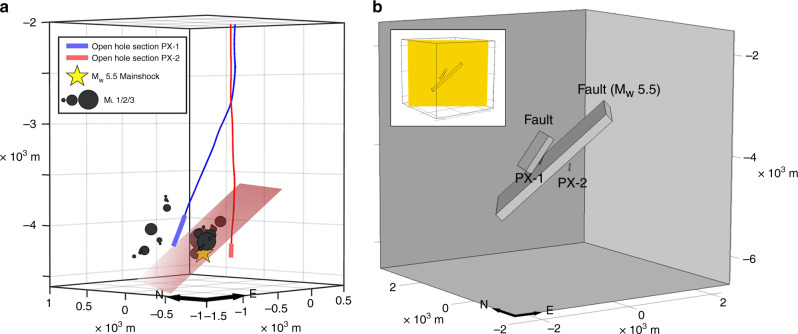
Pore pressure model setup. The model dimensions, locations of injection intervals of PX-1 and PX-2, and two faults identified by seismicity are included. **a** Shows earthquake locations relative to PX-1 and PX-2 as well as the M_w_ 5.5 fault plane (red rectangle). **b** Model dimensions and fault locations. Inset shows cross-section location (yellow rectangle) for main part of figure and results in Fig. 5^21^.

Multiple observations corroborate the existence of an inclined fault between PX-1 and PX-2^21,27^. The drilling report for PX-1 indicates mud loss over 4082–4181 m depth interval, which suggests that a higher permeability damage zone intersects the well. The drilling report for PX-2 shows the largest amount of mud loss occurred from 3804 to 3830 m. Leaking occurring at shallower locations in PX-2 and deeper locations in PX-1 suggests an inclined permeable zone, or fault damage zone, intercepting both wells. Logging data showed that fault gouges were present at depths of 3785–3805 m in PX-2. A 3-day single well injection test in PX-1 showed a distinctly steep slope on the time versus water level increase curve. A steeper slope is characteristic of the existence of a flow barrier, such as a fault with a low permeability core impeding cross-fault flow from the injection well. Finally and most directly, seismicity relocations reveal a well-defined fault plane orientated N34E and dipping 43°NW (Fig. 4a) passing through PX-2 at ~3800 m depth^21^. Seismicity relocations also identified a smaller fault near PX-1 where hypocenters aligned along an approximately planar feature^27^.

Hydrologic properties of faults^28^ and effects of the presence of faults on induced seismicity^29–31^ has been a subject of research for decades. A widely used fault permeability architecture is a less permeable fault core bounded on both sides by more permeable damage zones^32^. Such fault permeability architecture has been applied to study injection-induced seismicity. Yehya et al.^33^ showed that high-permeability fault damage zones facilitate diffusion of elevated fluid pressures from injection to greater distances and deeper locations. Fault zones at regional scale can be more complex with multiple basic fault architectures such as those in southeastern Korea^34^. Sutherland et al.^35^ inferred the existence of an alteration zone imbedded between the low permeability core and high permeability damage zones and that hydrologic processes in this zone affect fault strength and seismic properties. The basic physics is that a low permeability core is prone to thermal pressurization during slip^36,37^, which weakens the fault and leads to seismic radiation. The seismicity then causes permeability to increase and fluid flow to be enhanced in the alteration zone, which then prepares the fault for future ruptures.

Ree et al.^38^ carried out permeability tests on samples of a fault exposed to the north and south of the Pohang EGS site. These fault materials are the same age as the basement at the Pohang EGS site. Their study suggested that hydraulic conductivity of the fault gouge core and brecciated damage zone are 10^−13^–10^−12^ and 10^−6^–10^-4^ m s^−1^, respectively. Based on these results, hydraulic diffusivity of 10^−6^ m^2^ s^−1^ for the fault core and 0.1–1.0 m^2^ s^−1^ for the damage zone were used in the pore pressure model.

### Pore pressure modeling

We constructed a three-dimensional model with a domain of 5.0 km × 5.0 km × 5.0 km and two injection wells, PX-1 and PX-2, near the domain center (Fig. 4b). This domain size ensures that pore pressure change around the injection wells will not reach model boundaries, therefore any uncertainty in applied boundary conditions will not affect modeled pore pressure change results. No-flow boundary conditions are applied to all sides of the model domain. Wellhead pressure data are applied at the open interval of PX-1 and PX-2 as external perturbations to the system. Frictional loss of pressure along well casing was estimated to be negligible.

Using the hydraulic diffusivities and wellhead pressure data as input and addressing the uncertainties in fault permeability through sensitivity studies (see Methods section), we developed two model scenarios to assess pore pressure changes and their relation to seismicity. As described in the Methods section, we conducted extensive model calibrations of hydraulic diffusivity against field data of injection rate/volume and wellhead pressure. Model calibration resulted in two best-fit parameter scenarios, which are implemented as Case A and Case B. Case A is a fault model that includes two identified faults. The first fault has a 10 m-thick low-permeability core bounded on both sides by an 85 m-thick high-permeability damage zones between PX-1 and PX-2. The second fault is a smaller 130 m-thick high-permeability fault near PX-1. Orientations of the faults are based on seismicity relocations. Case B is a high-permeability fault case and similar to Case A except that the first fault does not include a low-permeability core. We consider Case A the best approximation of the actual system and a preferred model, while Case B addresses uncertainty about the existence of a fault core. Background diffusivity for both cases is 0.01 m^2^ s^−1^. Case A with a low-permeability fault core and Case B without represent end-members of the subsurface system (Table 1).

The overall state of the pore pressure regime can be considered as superposition of a hydrostatic background condition and changes caused by any fluid stimulation activities. Because we seek to calculate pore pressure change due to applied pressure during hydraulic stimulation, we set initial pore pressure change as zero. We used commencement of the first stimulation as the starting point for the model, and model results presented here only reflect pore pressure changes related to the five hydraulic stimulations.

### Spatial pore pressure change

**Fig. 5:**
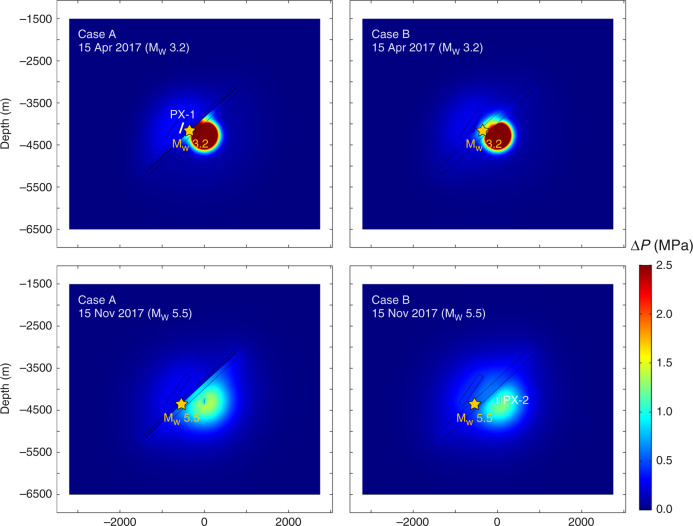
Modeled pore pressure change. The pore pressure change (MPa) is shown in vertical cross-section (location shown in inset in Fig. 4b) through PX-1 and PX-2 on April 15, 2017 (upper row) and November 15, 2017 (lower row) when the M_w_ 3.2 and the M_w_ 5.5 earthquakes occurred, respectively. Left and right columns correspond to Cases A (core hydraulic diffusivity *D* = 1.0 × 10^−6^ m^2^ s^−1^ and damage zone *D* = 0.1 m^2^ s^−1^, small fault *D* = 1.0 m^2^ s^−1^) and Case B (same as Case A except without a fault core).

Numerical modeling was conducted for two cases described above. Two time snapshots of spatial distributions of pore pressure change in cross-section are presented in Fig. 5 where the upper row is on April 15, 2017 and lower row is on November 15, 2017, when the M_w_ 3.2 and the M_w_ 5.5 earthquakes occurred. Panels on the left are for Case A and right for Case B.

In both Cases, pressure changes exceeded 0.01 MPa around the injection locations on April 15, 2017. Pressure change around PX-1 is largely the result of the relatively strong second stimulation that took place in PX-1 4 months earlier in December 2016 (Fig. 2). Pressure change around PX-1 area suggests that pore pressure perturbations from hydraulic stimulation can persist for months and longer. Pressure change around PX-2 is directly related to the third stimulation that took place from March 16 to April 14, 2017 immediately preceding the M_w_ 3.2 earthquake. In Case B, pressure change in areas between PX-1 and PX-2 reflect superimposed effects of perturbations from both wells. As time progresses, pore pressure change overall increased (lower row, Fig. 5). As anticipated, the subsequent fourth stimulation in PX-1 and fifth stimulation in PX-2 added to the pore pressure change.

Two results are highlighted here. One is that spatial extent of pressure change expanded with time. The areal expansion, while not surprising, is noteworthy for induced seismicity because pore pressure increasing over a broader area boosts the likelihood of encountering and activating preexisting faults as relatively small changes in pore pressure could trigger fault slip^39,40^. The other is that the fault between PX-1 and PX-2 emerges as a key feature in pore pressure diffusion. The low-permeability fault core acts as a flow barrier that impedes pressure diffusion and fluid flow across the fault, and effectively separates the pore pressure field into two (Fig. 5). The high-permeability damage zones bounding the fault core, on the other hand, facilitate pressure diffusion along the fault, allowing the pressure influence to spread over an elongated narrow zone near the fault, which makes the fault and its nearby region more vulnerable to slip.

### Temporal pore pressure change at hypocenters

We note that in both Cases pore pressure change is near its maximum value when the M_w_ 3.2 event occurred on April 15, 2017 (Fig. 5). The M_w_ 3.2 hypocenter is located within the fault damage zone on the PX-2 side. On April 15, 2017, pore pressure change at the hypocenter vicinity is 0.15–0.25 MPa, which is above the cited threshold pore pressure change of 0.01 MPa for triggering seismicity on critically stressed faults^40,41^. Stress analyses at the Pohang EGS site showed that the fault was indeed critically stressed^21^. The temporal variation patterns suggest that pressure change at the time of the earthquake is the result of the first and third stimulations in PX-2. Stimulations in PX-1 had little influence on pore pressures in the M_w_ 3.2 hypocenter area primarily because the fault with a low-permeability core impedes cross-fault pore pressure diffusion. For Case B that has two permeable faults without a low-permeability fault core, pore pressure change at this location responded to not only stimulations in PX-2 but also stimulations in PX-1. Comparing with Case A, the overall smaller values in Case B are due to absence of a low-permeability fault core allowing pressure diffusion easily over a larger area (Fig. 5).

The fourth stimulation was conducted in PX-1 from August 7 to 14, 2017, and the fifth in PX-2 from August 30 to September 18, 2017. On November 15, 2017, the M_w_ 5.5 earthquake occurred on the same fault plane as the M_w_ 3.2 event but further down dip (Fig. 4). Modeled pore pressure changes at this hypocenter in both Case A and Case B are smaller because it is farther from the injection locations (Fig. 5a). Although modeled pore pressure change at the time of the earthquake was not at its maximum, it was elevated at 0.05–0.08 MPa. The source of this pressure change is the third and fifth stimulations in PX-2.

### Coulomb static stress transfer modeling

**Fig. 6:**
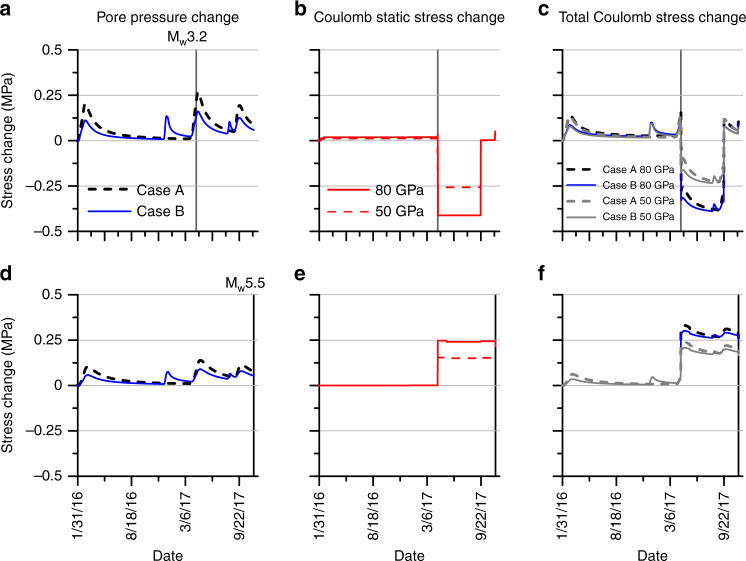
Pore pressure and Coulomb static stress transfer modeling. The model results are for the M_w_ 3.2 foreshock location (top row, **a**–**c**) and the M_w_ 5.5 mainshock location (bottom row, **d**–**f**). Pore pressure modeling results (**a**, **d**) for Case A (black dashed) and Case B (blue). Coulomb static stress transfer modeling results (**b**, **e**) at each location using a Young’s modulus of 80 GPa (red) and 50 GPa (red dashed). Total Coulomb stress change (**c**, **f**) is calculated by adding modeled pore pressures multiplied by a coefficient of friction of 0.6 and the Coulomb static stress change. Results for modeling using a Young’s modulus of 80 GPa is in blue and dashed black; modeling using a Young’s modulus of 50 GPa is in gray. The M_w_ 3.2 event is marked in the first row by a vertical gray solid line and the M_w_ 5.5 event is marked in the second row by a vertical black line. Source data are provided as a Source Data file.

Coulomb static stress transfer was calculated for 59 relocated earthquakes^27^ with M_w_ > 0.3. Using the focal mechanisms calculated by Ellsworth et al.^27^, the cumulative static Coulomb stress change was calculated at each event location to determine the spatiotemporal evolution of stress change. Static Coulomb stress transfer was calculated using two values of Young’s modulus (50 and 80 GPa). Using a Young’s modulus of 50 GPa, 39 of the 59 modeled events (66%) occurred in areas of positive Coulomb static stress change and 27 (46%) occurred in areas where the Coulomb static stress change exceeded 0.01 MPa which is the cited threshold for triggering^40,41^. Using a Young’s modulus of 80 GPa, 42 (71%) events occurred in areas of positive Coulomb static stress change and 32 (54%) occurred in areas where the static stress change exceeded 0.01 MPa. The calculated Coulomb static stress change at the locations of the M_w_ 3.2 foreshock and the M_w_ 5.5 mainshock at the time of each event were 0.02 and 0.13 MPa, respectively (0.04 and 0.15 MPa for modeling with a Young’s modulus of 80 GPa) (Fig. 6b, e). Decrease or increase of Coulomb static stress at any location depends on its relative location to the hypocenter and the focal mechanism of the source earthquake. The large increase of Coulomb static stress at the location of the M_w_ 5.5 event is directly correlated to the occurrence of the M_w_ 3.2 foreshock (Fig. 6e). An important point to note is that the Coulomb static stress change at the location of M_w_ 5.5 event exceeded the modeled pore pressure change of 0.05–0.08 MPa. Translating the modeled pore pressure change to the Coulomb stress with a fault friction coefficient of 0.6^21^ and adding it to the Coulomb static stress transfer estimates, we obtained the total Coulomb stress change (Fig. 6c and f). The range of total Coulomb stress increase at the M_w_ 3.2 location at the time of the earthquake was 0.09–0.16 MPa and at the M_w_ 5.5 location was ~0.16–0.20 MPa. The Coulomb static stress transfer more than doubles the stress change at the location of the mainshock in comparison to pore pressure increase alone. Data for Coulomb modeling and full results are presented in Supplementary Table 1.

## Discussion

Constrained by the Pohang field data, we conducted two separate models. The pore-pressure modeling showed that pore pressure changes are sufficient to induce seismicity prior to the mainshock. The Coulomb stress modeling demonstrated that the static stress change played a major role in triggering the mainshock. While the two models are not physically linked, the emphasis here is to examine the respective contributions from pore pressure and from Coulomb static stress change to induce seismicity. For a frictional coefficient 0.6, modeled pore pressure change of 0.05–0.25 MPa translates to a strength reduction, or a Coulomb stress increase, of 0.03–0.15 MPa. This strength reduction range exceeds the cited threshold of 0.01 MPa^40,41^ for inducing slip on critically stressed faults. While pore pressure changes could be sufficient, existence of a critically stressed fault that is optimally aligned for failure is also necessary for induced seismicity to occur. The case study also indicates that pore pressure change is a time sensitive variable, while prior stress and fault conditions are static in the timeframe of interest. Once pore pressure diffusion is set in motion, pore pressure and rock stress interactions start to play a role through gradually reducing fault strength^14^. Additionally, injected fluids mix with formation fluids near the injection well and short distances away from the well, but formation fluids under the induced pressure gradient may seep into and weaken faults at much greater distances^42^.

Once initial seismicity is induced, Coulomb stress transfer can play a role in the stability of faults in affected areas^17,19,20,41^. With seismicity occurring on a fault, static Coulomb stress transfer will follow, which may bring the fault closer to failure as indicated by the 66–71% of events that had Coulomb static stress transfer modeling occurring in areas of positive Coulomb stress change. As pore pressure diffusion continues, more seismicity could occur. These processes can decrease effective stress, increase differential stress, reduce the coefficient of friction, and weaken the fault.

One source of recurring doubt on whether an earthquake is induced is time delay between injection activity and mainshocks. More importantly, there still lacks a physical explanation for delays. In this Pohang example, time lag between the last stimulation and the M_w_ 5.5 mainshock was 2 months. Because the area influenced by pore pressure change continues to expand with time after the stimulation ceased, as pore pressure diffusion continues over a broader area. Thus, the chance for elevated pore pressure to encounter preexisting and critically stressed faults is enhanced. Consequently, seismicity can occur. The same physics behind the time delay also helps explain reported spatial separation between seismicity and injection laterally or in depth^43^. Even when injection stops, pore pressure diffusion will continue. While a mainshock could happen when pore pressure is at its peak, it could also happen before or after peak pore pressure. In the case of Pohang, the M_w_ 3.2 occurred just before peak pore pressure and the M_w_ 5.5 occurred after peak pore pressure at the respective hypocenters. Identifying a progressively weakened fault in time may be the key to foreseeing unintended major shocks. When earthquakes lineup, they often reveal pre-existing faults and more earthquakes could follow if injection continues, therefore, injection should cease. We note that stopping injection has not always prevented earthquakes and in some cases larger events from occurring, thus, temporal delays of seismicity should not be overlooked. Ellsworth et al.^27^ found that the magnitude of the Pohang mainshock conflicts with the injection volume–magnitude relation of McGarr^44^ but is consistent with the analysis of van der Elst et al.^45^ and Galis et al.^46^. Research on induced seismicity magnitude is ongoing through relating stressing rate to seismicity rate then relating probabilistic seismicity frequency to magnitude^47,48^. Moreover, the length of the fault revealed could be useful for estimating future earthquake magnitudes.

**Fig. 7:**
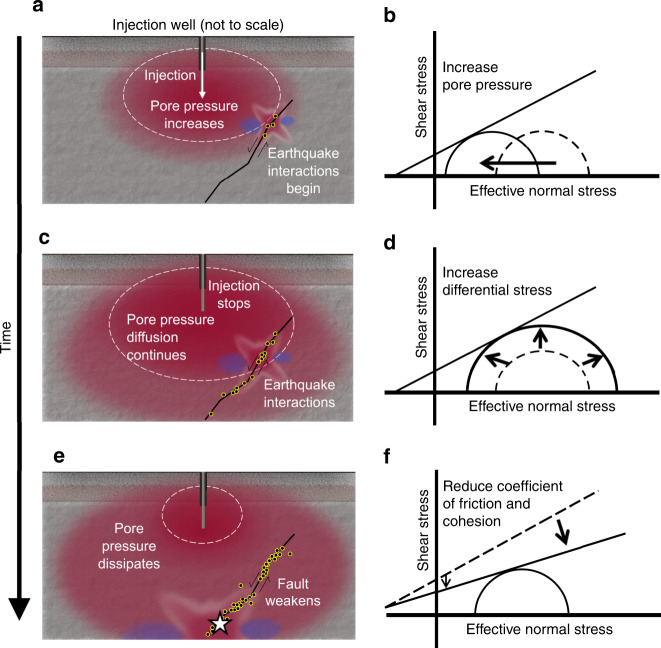
Conceptual illustrations of causal mechanism. Red areas represent pore pressure increase from fluid injection. Superimposed on the pore pressure cloud is the Coulomb static stress. The black line is a fault with sense of slip shown with arrows and earthquakes are indicated by small dots outlined in yellow. Dashed white contour is a pore pressure contour of constant magnitude. Elapsed time since start of injection increases from first to third row. **a** Pore pressure increase after a period of constant injection with the start of induced seismicity. **b** Mohr–Coulomb diagram showing triggering due to increased pore pressure along a preexisting critically stressed fault. **c** Pore pressure increase continues to expand following cessation of injection and more earthquakes are induced. **d** Mohr–Coulomb diagram showing triggering due to increase of differential stress along the fault. **e** High pore pressure increase starts to dissipate, but moderate pore pressure increase continues to diffuse away from well, and earthquakes continue and a larger event occurs (white star). **f** Mohr–Coulomb diagram showing triggering caused by weakening of the fault due to reduction of the coefficient of friction and reduced cohesion^42^.

It has been reported that the largest events have occurred in the post-injection period at other EGS sites^2,5^ and numerous wastewater injection sites^14,49–51^. Research is on-going to understand the mechanism of why and how post-injection earthquakes occur, particularly at the wastewater injection induced seismicity area. Poroelastic stress transfer^14^, aseismic creep^15,16^, density-driven pressure transients^52^, and static Coulomb stress change^17,18,20^ are some of the mechanisms proposed. This study adds to the on-going research to better understand the phenomena that largest events can occur after the injection ceases. On the basis of the Pohang case study and building on knowledge from prior studies, we present a mechanism for causation of injection-induced seismicity that for the first time integrates pore pressure and Coulomb static stress transfer in a unified framework. Fluids injected under pressure initiate pore pressure diffusion, causing pore pressure to increase from the injection locale outwards and triggering seismicity on pre-existing critically stressed faults (Fig. 7a, b). As injection continues, the region influenced by elevated pore pressure expands through time and more seismicity is triggered (Fig. 7c). Meanwhile the cumulative seismicity increases static Coulomb stress on the fault as illustrated by the enlarged Mohr circle (Fig. 7d) because of increased differential stress and weakens the integrity of the fault (Fig. 7f). As earlier seismicity reactivates patches of the fault, the integrity of the fault is damaged by tensile and shear, thus the cohesion of the fault may be reduced (Fig. 7f). Faults frictional properties are known to be slip rate dependent^53,54^ and slip weakening was observed in field studies^15,16^. Weakening can be caused by losing contact area and old adhesive contacts being replaced by less adhesive younger contact^53^. Slip-induced thermal pressurization in low-permeability faults is also suggested to weaken the fault^36,37^. As a result, a larger event could happen on repeatedly partially activated faults even when injection stops (Fig. 7e).

The role of fluids and pore pressure in faulting has long been recognized such as Hickman et al.^55^ and Sibson^56^. Here, we investigated mechanisms beyond pore pressure increase by combining pore pressure with Coulomb stress calculations using actual seismicity data. This study demonstrated that pore pressure buildup from injection was sufficient to initiate seismicity on a critically stressed fault and these initial earthquakes could generate additional Coulomb static stress that can exceed the pore pressure change values. In conclusion, we propose a multi-process causal mechanism for injection-induced earthquakes as follows. Pore pressure increase and earthquake interactions lead to fault weakening and ultimately triggering larger earthquakes later in the process. We suggest that it is prudent that pore pressure change, initial seismicity locations, and Coulomb static stress transfer from seismicity earlier in the sequence are assessed in real-time. Establishing local seismicity monitoring networks and conducting relocation analysis allows early identification of preexisting critically stressed faults. Coulomb static stress transfer analysis provides valuable information on stress redistribution. While direct in-situ pore pressure observations are limited and often logistically prohibitive, pore pressure modeling is a practical alternative, although uncertainties associated with model results need to be clearly understood.

## Methods

### Injection data

The Pohang EGS is to exploit geothermal energy at depths of >4 km in granitic bedrocks. Two exploratory wells, PX-1 and PX-2, were drilled to depths of 4215 and 4340 m, respectively. They are cased along their entire depths except for the bottom 313 m in PX-1 and 140 m in PX-2 that are open for fluid injection and flow back. The two wells are separated by 6 m on the ground surface and ~600 m at the bottomholes.

Five hydraulic stimulations were conducted in PX-1 and PX-2 from January 29, 2016 to September 18, 2017. The first, third, and fifth stimulations were conducted in PX-2 and the second and fourth in PX-1. Each hydraulic stimulation involved one period of injection when water is forced into the formation under a wellhead pressure and repeated periods of shut-in and water flow back to the surface. An M_w_ 3.2 event occurred immediately after the third stimulation in PX-2 and the M_w_ 5.5 event occurred 2 months after the fifth stimulation in PX-2.

Injection rates and wellhead pressures for all five stimulations were recorded. The temporal resolutions for these data are seconds for PX-1 and one minute for PX-2. Figure 2a shows the timeline of stimulations, M_w_ 3.2 and M_w_ 5.5 events, injection rates, and net injection volume over time. Wellhead pressures along with injection rates for five individual stimulations are shown in Fig. 2b–f. In PX-2, maximum wellhead pressure and injection rate reached 89.20 MPa and 46.83 × 10^−3^ m^3^ s^−1^ during the first stimulation (Fig. 2b). In PX-1, the maximum wellhead pressure and injection rate reached 27.71 MPa and 19.08 × 10^−3^ m^3^ s^−1^ during the second stimulation (Fig. 2c). One key observation from Fig. 2b–f is that the injection pressure was overall higher for PX-2 than that for PX-1 for similar injection rates.

### Pore pressure change modeling

Despite that this study is related to EGS where heat transport is inherently linked to fluid flow, our modeling focused on pore pressure only and the reasons are as follows. The M_W_ 5.5 Pohang earthquake took place during the early stage of the EGS development and the site was far from reaching the stage of circulating fluid between the two exploration wells. There was no fluid extraction at the site. Given the relatively short duration of 2 years from the first stimulation to the shut down and that thermal diffusivity is often several orders of magnitude smaller than hydraulic diffusivity, very limited heat transport is expected. Pore pressure diffusion through an isotropic and homogeneous porous medium can be mathematically described by the following differential equation:

1 $$\frac{\partial }{{\partial x}}\left( {K_x\frac{{\partial h}}{{\partial x}}} \right) + \frac{\partial }{{\partial y}}\left( {K_y\frac{{\partial h}}{{\partial y}}} \right) + \frac{\partial }{{\partial z}}\left( {K_z\frac{{\partial h}}{{\partial z}}} \right) = S_{\mathrm{{s}}}\frac{{\partial h}}{{\partial t}} + q$$

where *h* is hydraulic head (L) that is the sum of pressure head and elevation head. Since there is no change in elevation head when calculating only pressure change caused by hydraulic stimulation, the change of hydraulic head is equal to that of pressure head. Pore pressure is then calculated by multiplying the pressure head by the specific weight of water (*P* = *Ψγ*, where *P* is pore pressure, *Ψ* is pressure head, and *γ* is specific weight of water). *K* (L t^−1^) is hydraulic conductivity (in the *x*, *y*, and *z* directions) and *S*_S_ (L^−1^) is specific storage. The ratio of *K* and *S*_S_ is hydraulic diffusivity *D* (L^2^ t^−1^). *q* is fluid source rate (t^−1^). Equation (1) was solved using the COMSOL Multiphysics software^57^.

Input parameters to the model include model domain dimensions, hydrologic conditions on model boundaries, and rock hydrologic properties, namely hydraulic conductivity and specific storage or hydraulic diffusivity (Table 1). The physical model domain is discretized into 284,937 and 259,842 irregular elements for Case A and Case B respectively, with refined finer grids near the wells and the faults and coarser grids toward the model boundaries. For time-dependent problems, initial pore pressure condition is also required. Because we seek changes in pore pressure in response to hydraulic stimulations, the initial pore pressure changes can be set to zero. With these input parameters, the numerical model predicts pore pressure field changes through time subjected to hydraulic stimulations.

Either wellhead pressure as specified pressure or injection rate as a fluid source can be applied as the hydrologic perturbation to the model over the injection intervals. When wellhead pressures are applied, injection and flow back rates are used as model constraints. In addition, we estimated pressure loss due to friction along well casing using the Darcy–Weisbach equation^58^. These losses amount to ~1% of the wellhead pressure and we considered them negligible. For the three phases in each stimulation, wellhead pressure was applied during injection, pressure was set to zero during flow back, and no pressure was specified during shut-in so that any residual pressure from stimulation could attenuate according to the hydraulic properties of the rock formation.

### Use of stimulation data for inferring hydrologic properties

One of the main challenges in modeling pore pressure propagation to study injection and seismicity is the lack of in-situ test data for hydrologic properties, namely permeability (or hydraulic conductivity) and specific storage, or hydraulic diffusivity. This is because rock core samples are rare and access to the deep locations is limited. Dedicated multi-well pumping tests to obtain *K* and *S*_S_ for rock formations at such depths are also limited. This study utilized the hydraulic stimulation data, i.e., pressure change versus time over multiple periods to estimate hydraulic conductivity. The process of pressure build-up during injection and delay during flow back is an approximation to an in-situ single-borehole aquifer test. We note that there are limitations of single-well aquifer tests, and we were cognizant in interpreting the data. In the absence of direct aquifer test data, repeated hydraulic stimulations offered a valuable alternative for deriving hydrologic properties.

Analytical methods and numerical modeling can be applied to estimate hydraulic conductivity and hydraulic diffusivity using the wellhead pressure and injection rates. The analytical approach is the Jacob straight-line method^23^ that uses pore pressure change with time at an observation location during pumping. We note that the method of estimating hydraulic conductivity and specific storage was originally developed for multi-well systems, i.e., pumping from one well and observing drawdowns in another well away from the pumping well. The method has been extended to single-well systems^59^, but the method is not reliable for estimating *S*_S_ and therefore is only used for hydraulic conductivity estimation. The equations for hydraulic conductivity is as follows^23^:

2 $$K = \frac{Q}{{4\pi b\left( {s_2 - s_1} \right)}}{\mathrm{{ln}}}\frac{{t_2}}{{t_1}},$$

where *Q* is the pumping rate (L^3^ t^−1^), *b* is the open borehole length (L), *s*_1_ and *s*_2_ are pressure head drawdown (L) at time *t*_1_ and *t*_2_. We used the early part of every episode of pressure buildups and drawdown where the wellhead pressure versus log time is a straight line such that the key assumptions of the Cooper–Jacob equation are satisfied. Throughout the five stimulations, pressures experienced repeated build-up and drawdowns (Fig. 2).

To complement the analytical approach, we used numerical modeling to derive the parameter values (Table 1), using wellhead pressure as input and injection rate as constraints. We adjust hydraulic conductivity and specific storage values, so that modeled and measured changes in the flux rate and total net volume of fluid flux at the injection locations are consistent. We note that a complete match between the modeled and measured data is impractical because there are inconsistencies in reported data. For example, during a shut-in period in the second stimulation in PX-1, there were three wellhead pressure decrease–increase pulses (Fig. 3a). This is theoretically impossible and practically difficult to explain by any physics. In addition, the permeability could increase during the short time period of high-pressure injection. Sonnenthal et al.^60^ showed that fine tuning the permeability change after stimulation induced fracture shearing improved matching the modeled and observed flow rates and wellhead pressures. Because our data showed no sustained permeability enhancement over the study period, any fracture opening or shearing was likely short lived, therefore, we did not include such transient permeability changes in our model. We placed our calibration emphasis on low to medium wellhead pressure data to allow the model best represents the overall physical processes.

We note that aquifer properties, *K* and *S*_s_, therefore their ratio *D*, can be influenced by temperature. The reported temperature at the injection depth varied from 145 to 175 °C and the average temperature was 15 °C for injected water. Aquifer property differences due to changes in density, viscosity, and compressibility over this temperature range^61^ can lead to a *D* value at an average temperature of 150 °C being approximately four times higher than the value at 15 °C, this is considered a relatively small variation because hydraulic diffusivity generally varies over many orders of magnitude. The *K* values in this study were derived from measured pressure data, and, therefore, should reflect the in-situ condition at the injection depth. The compressibility values we used in computing *S*_s_ were from mechanical lab tests^24^. In addition, the *S*_s_ change estimated due to water compressibility change^61^ over the above-mentioned temperature range is minimal. Final diffusivity values, used for pore pressure modeling, were determined by calibrating the numerical model against the hydraulic stimulation and seismology data.

Figure 3 demonstrates examples of model calibration results. Using wellhead pressure as the input and modeled flow rate at the injection location is compared with the reported injection rate for the second stimulation in PX-1 (Fig. 3a) and the first stimulation in PX-2 (Fig. 3b). These two stimulations were chosen because they were free from later hydraulic interferences between PX-1 and PX-2. For PX-1, hydraulic conductivity from 5.0 × 10^−9^ to 1.0 × 10^−8^ m s^−1^ and specific storage from 1.0 × 10^−6^ to 1.0 × 10^−5^ m^−1^ produced more satisfactory matches between modeled and measured injection rate. The modeled net injection volume was larger than the measured one, which is largely because model overestimation during the shut-in periods using the reported wellhead pressure. For PX-2 (Fig. 3b), hydraulic conductivity from 1.0 × 10^−9^ to 5.0 × 10^−9^ m s^−1^ and specific storage from 5.0 × 10^−7^ to 5.0 × 10^−6^ m^−1^ produced better matches between modeled and measured injection rate. Calibration against measured injection rate and net injection volume narrowed the range of hydraulic diffusivity values to 5.0 × 10^−4^ to 1.0 × 10^−2^ m^2^ s^−1^, which is in good agreement with the only reported in-situ test value of 3.5 × 10^−4^ m^2^ s^−1^. This range was further narrowed to 1.0 × 10^−2^ m^2^ s^−1^ for the basement rock after the calibration against the seismicity analysis. When diffusivity is lower than 1.0 × 10^−2^ m^2^ s^−1^, there would be little pore pressure change at the hypocenters of the seismicity during hydraulic stimulations.

### Coulomb static stress transfer modeling

Of 93 relocated seismic events^27^, 60 events were M_w_ > 0.3 including the M_w_ 5.5 mainshock. We conducted Coulomb static stress modeling for 59 relocated events of M_w_ > 0.3 that occurred prior to the M_w_ 5.5 event. Using the USGS Coulomb 3.3 software^62,63^, the relocated events^21,27^, and the focal mechanisms^21,27^, we calculated the Coulomb static stress change at each subsequent event location and at the locations of the M_w_ 3.2 foreshock and M_w_ 5.5 mainshock. In order to calculate the Coulomb static stress change, fault parameters, such as fault length, width, and average slip are needed. We used the equations of Leonard^64^, which relates moment magnitude to these parameters and is applicable for smaller magnitude earthquakes. Fourteen of the relocated events only had local magnitudes (M_L_) calculated without moment magnitude (M_w_). Therefore, we used the 46 events with both M_L_ and M_w_ magnitudes calculated to estimate a relationship between the two magnitude scales through linear regression. The equation, M_w_ = 0.8827M_L_ + 0.4594, was used to convert local magnitude values to moment magnitude. Focal mechanisms were calculated by Ellsworth et al.^27^ for 47 of the 60 events over M_w_ 0.3. For the 13 events that have no reported focal mechanism, we used the focal mechanism of the event closest to each of the 13 events in the Coulomb static stress calculations. The most likely nodal plane was chosen for each event based on the regional stress^21,27^, seismicity patterns^21,27^, and linear slip inversion of Interferometric synthetic aperture radar (InSAR) data of the mainshock^65^.

We calculated the cumulative Coulomb static stress change caused by the previous events at the location of each subsequent event using the event’s focal mechanism as the receiver fault for the calculation. Since the calculations were made on specified fault orientations, the background regional stress was not used in the calculations. The coefficient of friction was set at 0.6, which is consistent with the value used in the models to calculate the focal mechanisms^27^. A Poisson’s ratio of 0.25 and two values of Young’s modulus (50 and 80 GPa) were used. Laboratory tests on core samples from the site indicated an average Young’s Modulus of 33.5 GPa while a larger Young’s modulus of ~45 GPa was measured using P- and S-waves^24^. The discrepancy between laboratory measurements and in situ measurements of rock properties is often recorded and can be attributed to stress-relief damage^66^ when the cores are extracted. Therefore, we modeled the static stress change using two end-member Young’s moduli. In addition, short-term permeability increase during stimulations may indicate a reduction in elastic modulus for rock volumes in the immediate vicinity of the open sections of the wells. Most seismicity, however, occurred on faults away from the permeability increasing area. Therefore, a direct connection between changing permeability and corresponding change in mechanical property cannot be established. It is unclear how much, if any, change mechanical properties experience in response to stimulations. We acknowledge that the static nature of the mechanical property used in our static Coulomb stress modeling is an approximation and representative of the bulk materials of the site. Model input data are included as Supplementary Data 1 and information about the execution of the model is included in Supplementary Note 1.

## Supplementary information

Supplementary Information

Description of Additional Supplementary Files

Supplementary Data 1

## Data Availability

The data that support the findings of this study are available from the corresponding author upon reasonable request. The seismicity data is available in the referenced citations. The source data underlying Figs. 2, 3, and 6 are provided as a Source Data file. Map outlines for Fig. 1a were downloaded from www.GADM.org for each country on June 7, 2019.

## Source Data

Source Data

## Code availability

The inputs for Coulomb static stress transfer modeling is included in the Supplementary Information and the Source Data. Coulomb 3.3 is a MATLAB application available from the USGS (https://earthquake.usgs.gov/research/software/coulomb/).
