# Supplementary material for: Causal mechanism of injection-induced earthquakes through the Mw 5.5 Pohang earthquake case study
Source: Nat Commun. 2020 May 26;11:2614. doi: 10.1038/s41467-020-16408-0 (PMC7251101; doi:10.1038/s41467-020-16408-0)
Supplement: Supplementary file 1 — Supplementary Information [file 41467_2020_16408_MOESM1_ESM.pdf]

Causal mechanism of injection-induced earthquakes through the M_w 5.5 Pohang earthquake case study

Yeo et al.

Supplementary Table 1: Coulomb modeling data and results

Origin time (MM/DD/YY hh:mm:ss.sss)	Event Location			Magnitudes		Focal Mechanism Nodal Plane Used			Coulomb Static Stress Results (Young's modulus = 80 GPa)			Coulomb Static Stress Results (Young's modulus = 50 GPa)		
	Latitude	Longitude	Depth (km)	Local Magnitude (M_L)	Moment Magnitude (M_w)	Strike	Dip	Rake	Coulomb Results at M_w 3.2 Location (MPa)	Coulomb Results at M_w 5.5 Location (MPa)	Coulomb Result at each event location (MPa)	Coulomb Results at M_w 3.2 Location (MPa)	Coulomb Results at M_w 5.5 Location (MPa)	Coulomb Result at each event location (MPa)
11/30/2015 03:52:20.35	36.1091	129.3768	3.96	0.80	0.97	275.1	68.6	35.9	NA	NA	NA	NA	NA	NA
02/04/2016 03:55:45.64	36.1087	129.3757	4.09	0.55	0.94	206.5	56.0	94.7	1.13E-04	2.00E-05	5.14E-04	5.83E-05	1.25E-05	3.21E-04
02/04/2016 19:09:52.44	36.1088	129.3760	4.10	0.69	1.07	218.4	37.8	137.0	1.89E-04	3.82E-05	7.95E-03	1.18E-04	2.38E-05	4.97E-03
02/06/2016 05:11:31.03	36.1070	129.3759	4.06	0.70	1.17	219.5	38.3	139.8	3.77E-04	3.73E-05	1.09E-03	2.36E-04	2.33E-05	6.86E-04
02/06/2016 15:01:33.72	36.1087	129.3759	4.08	0.70	1.08	214.6	50.1	115.0	9.44E-04	7.90E-06	1.15E-01	5.90E-04	5.00E-06	7.17E-02
02/07/2016 22:04:12.28	36.1072	129.3750	4.11	1.14	1.62	207.9	57.8	156.4	1.14E-03	2.60E-05	-1.90E-02	7.13E-04	1.62E-05	-1.19E-02
02/07/2016 22:04:15.40	36.1071	129.3755	4.15	0.64	1.29	207.9	57.8	156.4	1.33E-02	2.84E-04	-5.98E-02	8.33E-03	1.78E-04	-3.74E-02
02/17/2016 07:43:44.02	36.1069	129.3752	4.07	0.67	1.09	212.8	53.1	151.9	1.84E-02	8.77E-05	1.02E-02	1.15E-02	5.48E-05	6.40E-03
02/18/2016 13:08:16.51	36.1083	129.3762	3.98	0.61	1.00	275.1	68.6	35.9	1.89E-02	8.27E-05	1.58E-03	1.18E-02	5.17E-05	9.89E-04
03/12/2016 07:25:46.75	36.1079	129.3752	4.03	0.76	1.17	344.7	34.5	93.8	1.91E-02	1.17E-04	3.85E-02	1.19E-02	7.32E-05	2.41E-02
08/22/2016 11:48:29.20	36.1083	129.3772	4.03	0.77	1.19	275.1	68.6	35.9	1.86E-02	1.27E-04	-1.05E-02	1.16E-02	7.93E-05	-6.58E-03
12/18/2016 18:43:44.36	36.1128	129.3718	4.27	0.82	1.47	234.0	54.2	150.6	1.85E-02	1.42E-04	-1.59E-04	1.15E-02	8.88E-05	-9.91E-05
12/19/2016 02:20:36.62	36.1115	129.3740	3.77	0.91	1.41	199.5	58.6	103.5	1.85E-02	1.60E-04	8.87E-04	1.16E-02	9.99E-05	5.62E-04
12/19/2016 05:18:48.71	36.1112	129.3738	3.97	0.37	0.79	31.0	52.2	152.2	1.84E-02	1.61E-04	-1.46E-03	1.15E-02	1.00E-04	-9.14E-04
12/19/2016 08:04:21.25	36.1117	129.3746	3.78	0.57	1.10	202.4	70.6	177.4	1.84E-02	1.63E-04	-5.85E-03	1.15E-02	1.02E-04	-3.66E-03
12/19/2016 22:02:34.40	36.1106	129.3747	3.83	0.67	0.58	223.5	36.2	138.9	1.85E-02	1.89E-04	6.16E-03	1.15E-02	1.18E-04	3.85E-03
12/20/2016 07:56:08.02	36.1117	129.3732	4.17	1.21	1.46	32.8	86.6	156.3	1.85E-02	1.97E-04	4.13E-03	1.16E-02	1.23E-04	2.58E-03
12/21/2016 00:40:23.34	36.1128	129.3707	4.33	0.93	1.35	326.7	52.5	-24.5	1.85E-02	2.23E-04	-3.86E-03	1.16E-02	1.40E-04	-3.69E-03
12/22/2016 00:53:58.26	36.1115	129.3740	3.73	0.75	1.41	196.1	49.2	139.3	1.85E-02	2.44E-04	1.23E-01	1.16E-02	1.52E-04	7.67E-02
12/22/2016 20:31:32.52	36.1126	129.3721	4.28	1.80	2.19	238.7	69.8	150.4	1.86E-02	3.41E-04	-4.15E-03	1.16E-02	2.13E-04	-2.60E-03
12/24/2016 08:45:26.54	36.1101	129.3750	3.79	0.64	1.06	224.0	36.4	139.6	1.86E-02	3.48E-04	4.14E-04	1.16E-02	2.17E-04	-2.37E-04
12/25/2016 06:59:49.96	36.1101	129.3742	4.04	0.92	1.04	218.0	68.4	139.6	1.87E-02	3.81E-04	4.56E-03	1.17E-02	2.38E-04	2.85E-03
12/28/2016 15:12:14.44	36.1126	129.3714	4.29	0.91	1.32	223.9	57.5	165.0	1.89E-02	4.10E-04	2.87E-02	1.18E-02	2.56E-04	-1.93E-02
12/29/2016 12:32:25.47	36.1130	129.3737	4.07	2.15	2.36	250.3	64.6	156.7	1.89E-02	4.23E-04	3.97E-03	1.18E-02	2.65E-04	2.33E-03
12/29/2016 16:50:28.51	36.1102	129.3744	3.76	0.31	0.92	210.3	42.4	134.8	1.95E-02	5.38E-04	-3.34E-02	1.22E-02	3.36E-04	-2.09E-02
04/08/2017 13:13:55.33	36.1085	129.3759	4.13	0.44	0.72	218.4	37.8	137.0	1.95E-02	5.62E-04	1.05E-01	1.22E-02	3.51E-04	5.08E-02
04/15/2017 01:17:58.02	36.1099	129.3756	3.99	0.57	0.97	218.0	68.4	139.6	1.94E-02	5.54E-04	1.18E-02	1.21E-02	3.46E-04	7.38E-03
04/15/2017 02:31:07.25	36.1070	129.3749	4.10	1.55	1.56	208.7	56.1	134.1	3.88E-02	5.62E-04	6.51E-01	1.21E-02	3.51E-04	4.07E-01
04/15/2017 02:31:12.99	36.1077	129.3746	4.15	3.27	3.29	214.5	58.3	128.0	3.61E-02	9.27E-04	3.61E-02	2.25E-02	5.79E-04	2.25E-02
04/15/2017 02:31:41.43	36.1072	129.3770	3.98	2.03	2.09	204.2	61.6	135.7	-4.08E-01	2.39E-01	6.54E-02	-2.55E-01	1.49E-01	4.09E-02
04/15/2017 02:32:06.35	36.1072	129.3759	4.05	1.45	1.74	210.3	63.7	139.9	-4.06E-01	2.39E-01	-8.52E-02	-2.53E-01	1.50E-01	-4.07E-02
04/15/2017 02:35:25.50	36.1070	129.3733	4.25	0.71	1.09	184.2	32.0	104.8	-4.04E-01	2.39E-01	2.29E-01	-2.52E-01	1.50E-01	1.38E-01
04/15/2017 02:36:30.21	36.1066	129.3726	4.25	0.37	0.79	213.7	56.8	140.5	-4.05E-01	2.43E-01	3.07E-01	-2.53E-01	1.52E-01	1.92E-01
04/15/2017 04:31:07.54	36.1052	129.3725	4.21	0.40	0.81	234.2	56.8	143.7	-4.05E-01	2.48E-01	2.17E-01	-2.53E-01	1.55E-01	1.36E-01
04/15/2017 05:12:40.05	36.1076	129.3760	4.01	0.47	0.88	210.3	63.7	139.9	-4.05E-01	2.49E-01	9.26E-01	-2.53E-01	1.55E-01	5.79E-01
04/15/2017 05:28:59.30	36.1081	129.3748	4.11	0.70	1.07	344.7	34.5	93.8	-4.04E-01	2.49E-01	-4.73E-01	-2.53E-01	1.55E-01	-2.96E-01
04/15/2017 08:16:47.03	36.1066	129.3722	4.27	2.06	2.15	213.7	56.8	140.5	-4.13E-01	2.49E-01	2.34E-01	-2.58E-01	1.55E-01	1.47E-01
04/15/2017 09:47:59.70	36.1066	129.3753	4.03	0.45	0.86	212.8	53.1	151.9	-4.12E-01	2.47E-01	5.03E-01	-2.58E-01	1.54E-01	3.14E-01
04/15/2017 17:02:30.16	36.1058	129.3733	4.11	0.48	0.87	216.8	50.0	143.6	-4.12E-01	2.47E-01	3.55E-01	-2.58E-01	1.54E-01	2.22E-01
04/16/2017 01:44:46.80	36.1038	129.3691	4.20	0.97	1.55	249.0	66.7	162.7	-4.12E-01	2.47E-01	1.49E-02	-2.58E-01	1.54E-01	9.33E-03
04/16/2017 01:50:17.79	36.1040	129.3692	4.20	0.74	1.27	251.2	74.5	160.5	-4.12E-01	2.47E-01	-4.84E+00	-2.58E-01	1.54E-01	-3.03E+00
04/16/2017 09:41:37.46	36.1101	129.3768	4.10	0.35	0.77	218.4	37.8	137.0	-4.12E-01	2.47E-01	-1.03E-02	-2.58E-01	1.54E-01	-6.43E-03
04/20/2017 13:01:36.25	36.1037	129.3693	4.17	0.67	1.21	248.9	74.3	162.3	-4.12E-01	2.47E-01	7.39E-02	-2.58E-01	1.54E-01	4.62E-02
04/20/2017 22:14:20.48	36.1065	129.3746	4.07	0.66	0.76	216.2	50.2	138.1	-4.12E-01	2.47E-01	5.82E-01	-2.58E-01	1.54E-01	3.64E-01
04/21/2017 18:51:04.12	36.1052	129.3727	4.13	0.45	0.74	218.9	50.0	140.2	-4.12E-01	2.47E-01	1.24E-01	-2.57E-01	1.54E-01	7.77E-02
04/21/2017 23:58:10.32	36.1057	129.3729	4.13	0.96	1.26	208.5	40.7	145.7	-4.12E-01	2.47E-01	1.37E-01	-2.58E-01	1.54E-01	8.54E-02
05/18/2017 19:04:32.58	36.1048	129.3718	4.12	0.89	1.43	234.2	56.8	143.7	-4.12E-01	2.40E-01	5.67E-02	-2.57E-01	1.50E-01	3.54E-02
08/13/2017 21:42:36.88	36.1117	129.3734	4.21	0.67	1.21	302.9	70.1	-32.3	-4.12E-01	2.40E-01	5.76E-01	-2.57E-01	1.50E-01	3.60E-01
09/11/2017 07:19:24.37	36.1121	129.3751	3.86	1.54	2.03	223.0	57.6	145.3	-4.12E-01	2.40E-01	7.92E-02	-2.57E-01	1.50E-01	8.78E-02
09/15/2017 19:33:49.56	36.1060	129.3748	3.99	0.73	0.82	222.5	43.5	140.6	-4.11E-01	2.41E-01	1.79E-01	-2.57E-01	1.50E-01	1.12E-01
09/16/2017 08:55:55.76	36.1074	129.3737	4.09	1.78	1.68	218.9	45.0	140.2	-4.11E-01	2.41E-01	-1.63E-01	-2.57E-01	1.57E-01	-1.02E-01
09/22/2017 14:27:21.12	36.1080	129.3746	4.19	1.17	1.54	253.0	80.8	154.2	-4.33E-01	2.44E-01	-1.17E-01	-2.71E-01	1.53E-01	-7.37E-02
09/22/2017 14:27:41.34	36.1080	129.3743	4.21	0.77	1.27	253.0	80.8	154.2	-3.36E-02	2.44E-01	2.81E-02	-2.10E-02	1.52E-01	1.75E-02
09/22/2017 18:09:55.16	36.1077	129.3738	4.24	1.33	1.58	331.3	67.2	-4.2	2.97E-03	2.44E-01	-2.31E-02	1.86E-03	1.52E-01	-1.44E-02
11/14/2017 19:55:15.00	36.1056	129.3727	4.12	1.60	1.67	248.5	77.1	-178.5	4.80E-02	2.41E-01	1.45E+00	3.00E-02	1.51E-01	9.04E-01
11/14/2017 20:04:17.00	36.1054	129.3727	4.12	1.70	1.78	235.8	51.7	153.7	4.79E-02	2.41E-01	8.27E-02	3.00E-02	1.50E-01	-4.81E-02
11/14/2017 20:59:37.64	36.1055	129.3730	4.10	1.25	1.57	233.8	62.9	141.9	4.79E-02	2.37E-01	1.22E-01	2.99E-02	1.48E-01	7.65E-02
11/15/2017 05:22:32.19	36.1055	129.3729	4.09	2.12	2.20	224.1	57.7	137.6	4.79E-02	2.37E-01	-7.12E-01	2.99E-02	1.48E-01	-4.45E-01
11/15/2017 05:22:43.57	36.1054	129.3722	4.15	2.86	2.72	234.2	56.8	143.7	4.87E-02	2.15E-01	1.54E-01	3.04E-02	1.34E-01	9.68E-02
11/15/2017 05:29:31.33	36.1061	129.3726	4.27	5.35	5.56	213.7	51.2	128.4	5.17E-02	4.77E-02	4.77E-02	3.23E-02	2.98E-02	2.98E-02

Calculated moment magnitudes

Focal mechanism nodal plane from closest event

Supplementary Note 1

CODE INFORMATION READ ME

System Requirements

Software required:
Coulomb 3.3 which is open source from the USGS (https://earthquake.usgs.gov/research/software/coulomb/) is required.
Coulomb 3.3 runs on MATLAB 7.4 or higher.

Software dependencies and operating systems:

Quoted from: Toda, Shinji, Stein, R.S., Sevilgen, Volkan, and Lin, Jian, 2011, Coulomb 3.3 Graphic-rich deformation and stress-change software for earthquake, tectonic, and volcano research and teaching—user guide: U.S. Geological Survey Open-File Report 2011–1060, 63 p., available at https://pubs.usgs.gov/of/2011/1060/.

"Macintosh OSX, Windows PC, or UNIX computer
• MATLAB 7.4 or higher is required. There are a few MATLAB functions that work on PCs but not Macs. MATLAB 7.5 is not advised; it is full of bugs; there are strain problems with MATLAB 7.4.
• A color monitor of at least 600 x 400 pixel resolution. A laptop screen is fine.
• A text editor to modify ascii input files, and a spreadsheet to read tab-delimited text files. Some output files are created as .csv (Excel-friendly) files. Use a text editor that allows you to distinguish spaces from tabs, such as BBEdit (BBEdit Lite is free from http://www.barebones.com/) or Notepad on a Windows PC. Use a nonkerning (uniform-spacing) font, such as Monaco, so that numbers stay aligned."

Versions software has been tested on:
Windows PC; MATLAB R2019b (academic license)

Installation Guide:

MATLAB requires a license for use. Download Coulomb 3.3 from the website listed above and extract files.
Installation time is minimal depending on internet speed.

Demo/Instructions for use

Event data are included in the Coulomb results file (Coulomb_Results.xls)included in the supplementary information.

The input file (Coulomb_Input.inp) for Coulomb 3.3 used including all earthquakes modeled is included in the data source files.
Run Coulomb 3.3 in MATLAB and load this file. Under Functions -> Stress choose Coulomb Stress Change. Choose specified faults and input the strike, dip, and rake of the receiver fault. (In this case the mainshock focal mechanism: 213.7, 51.2, 128.4). Choose Calc. & View at the bottom right of the Stress control panel. An output file called dcff.cou is generated with the results at the depth indicated in the input file/Stress control panel. Run time will be several minutes.

To run for each individual event, the input file needed to be updated by removing the event of interest and all events after it. Update the #fixed value to match the number of events being modeled. Also update the DEPTH to the depth of the event of interest. After inputing the file as explained above, use the strike, dip, and rake of the event of interest. To change the Young's modulus, adjust the E1 and E2 values in the input file to the desired value.
