# Supplementary material for: Causal mechanism of injection-induced earthquakes through the Mw 5.5 Pohang earthquake case study
Source: Nat Commun. 2020 May 26;11:2614. doi: 10.1038/s41467-020-16408-0 (PMC7251101; doi:10.1038/s41467-020-16408-0)
Supplement: Supplementary file 2 — Description of Additional Supplementary Files [file 41467_2020_16408_MOESM2_ESM.pdf]

Title: Supplementary Data 1

Description: Input data for the Coulomb static stress transfer modeling,
